# Like, comment, subscribe: How journal editors can navigate social media competing interests

**DOI:** 10.3399/BJGPO.2023.0038

**Published:** 2023-03-14

**Authors:** Patrick Burch, Daniel Butler, Hajira Dambha-Miller

**Affiliations:** 1 Centre for Primary Care, University of Manchester, Manchester, UK; 2 Queen’s University Belfast, Belfast, Northern Ireland; 3 BJGP Open, Royal College of General Practitioners, London, UK

**Keywords:** editors, social media, ethics

Social media is an important tool for researchers, publishers, and doctors alike. In an era of ever-increasing connection, access to open social media platforms allow users to interact with potentially millions of others in a public arena on wide-ranging subjects with the purpose of gathering information, formulating opinions, persuading people, or simply passing the time by ‘scrolling vertically’. Social media platforms are, by default, publicly visible. Carefully formulated text alongside informal, seemingly innocuous contributions have the potential to create opportunities for momentary connections or conflict through comments, likes, and blocks. These moments are stored in perpetuity, creating potential for future conflict, as the internet never forgets.

So, how do journal editors navigate competing interests within social media in an era of ever-increasing connection? Editors are often social media platform users. Like everyone else, they may engage in current and previous interactions with authors, fellow editors, advisory members, or public representatives. Given the number of potential social interactions stored over time from account inception, should each of these individual interactions be declared as a competing interest by the editor? While there is a general acceptance and associated guidance from the Committee On Publication Ethics suggesting that those involved in medical publishing should declare competing interest that may directly affect, or be perceived to affect, decisionmaking processes,^1^ this is challenging to operationalise with social media interactions; the average social media user spends about 5 hours per month on Twitter and 23.7 hours per month on YouTube,^2^ before taking into account Instagram, Tik-Tok, or other social media platforms. Over time the average medical editor may have had thousands of social media interactions. Should each of these interactions be declared by the editor as a competing interest that may impact current and future manuscript decisions?

This is far more complex that declaring a financial conflict, which is relatively unambiguous. A non-financial competing interest has a range of definitions; at its broadest, this includes any aspect of an individual that could be perceived as potentially influencing a decisionmaking process. An editor deciding the outcome of an article submitted by a close friend is an obvious example. What about an editor who previously liked a tweet by a submitting author? Or an editor who may have supported a political view that contradicts an author view on social media? Can an editor impartially review a manuscript on eczema if they and the authors express different political views on, say, the pro-life versus pro-choice debate on Twitter? Does previous social media interaction — no matter how long ago — bias the editor’s judgment with regards all future manuscripts, irrespective of the subject matter?

Surely, sensibilities would suggest that there is a limit to how far this can and should be taken. As Rodwin argues, intellectual conflict is widespread and inherent to life.^3^ Recognising, publicly declaring, and attempting to manage every potential non-financial source of bias for journal editors would be an impossible task. It would also be a disproportionate infringement of privacy to ask editors to formally make public all their known personal characteristics, career ambitions, personal ambitions, or political viewpoints that could possibly be conceived of as influencing their decisionmaking. There is an element of confidence and trust required in the robustness of editorial processes, if this is the accepted standard for scientific publication. Most manuscripts will go through two independent external peer reviews, or if a desk-reject it may have received extensive interrogation between editorial board members and associate editors. Nowadays, most journals have moved towards collective decisionmaking and away from decisions by sole editors, thus reducing the potential impact of any individual competing interest. In manuscript meetings where editorial teams make decisions, it would be extremely time-consuming and arguably impossible to keep track of all potential past and future social media interactions for each person in the room.

While not all possible sources of bias need to be declared or actively managed, editors should be aware that social media creates an avenue for publicly displaying their potential biases. Some organisations, such as the British Broadcasting Corporation (BBC), have social media policies restricting staff from airing certain personal opinions.^4^ Our view is that the imposition of such guidance in medical publishing would be disproportionate and overly restrictive of personal freedoms, and has the potential to stifle academic debate. It would also be ineffectual, merely hiding potential biases rather than eliminating them. That is not to say journal editors should be unmindful of the impact of their public views on author submission. If editors state a personal view on social media, then they need consider how it may be perceived as a potential competing interest, and the concept of declaring and managing what a 'reasonable reader' might consider a competing interest is a still a sound one. It is doubtful that the inclusion of an 'opinions expressed' disclaimer in social media profiles will shield editorial staff from scrutiny or accusations of bias. All journals should state their competing interest policy, and any disputes or queries relating to it should be resolved by an independent reviewer. Ultimately it is up to authors where they choose to submit their manuscripts. There is no shortage of scientific journals and where competing interests are not managed, authors will simply go elsewhere.
